# *Omics* for Investigating Chitosan as an Antifungal and Gene Modulator

**DOI:** 10.3390/jof2010011

**Published:** 2016-03-03

**Authors:** Federico Lopez-Moya, Luis V. Lopez-Llorca

**Affiliations:** Laboratory of Plant Pathology, Multidisciplinary Institute for Environmental Studies (MIES) Ramon Margalef, Department of Marine Sciences and Applied Biology, University of Alicante, E-03080 Alicante, Spain

**Keywords:** chitosan, transcriptomics, genomics, gene modulator, antifungal, biocontrol fungi (BCF)

## Abstract

Chitosan is a biopolymer with a wide range of applications. The use of chitosan in clinical medicine to control infections by fungal pathogens such as *Candida* spp. is one of its most promising applications in view of the reduced number of antifungals available. Chitosan increases intracellular oxidative stress, then permeabilizes the plasma membrane of sensitive filamentous fungus *Neurospora crassa* and yeast. Transcriptomics reveals plasma membrane homeostasis and oxidative metabolism genes as key players in the response of fungi to chitosan. A lipase and a monosaccharide transporter, both inner plasma membrane proteins, and a glutathione transferase are main chitosan targets in *N. crassa.* Biocontrol fungi such as *Pochonia chlamydosporia* have a low content of polyunsaturated free fatty acids in their plasma membranes and are resistant to chitosan. Genome sequencing of *P. chlamydosporia* reveals a wide gene machinery to degrade and assimilate chitosan. Chitosan increases *P. chlamydosporia* sporulation and enhances parasitism of plant parasitic nematodes by the fungus. *Omics* studies allow understanding the mode of action of chitosan and help its development as an antifungal and gene modulator.

## 1. Introduction

Chitin is a key structural component of the exoskeleton of invertebrates and fungal cell walls. Its deacetylated form, chitosan, a polycation, permeabilizes the fungal membrane in an energy-dependent manner [1]. Membrane composition determines chitosan sensitivity in fungi [2]. Chitosan kills or compromises the growth of plant [3] and human fungal pathogens [4,5,6]. On the contrary, biocontrol fungi (BCF) such as nematophagous and entomopathogenic fungi are resistant to chitosan [7]. Perhaps as a result of coevolution with their hosts, these fungi have evolved chitosan-resistant low-fluidity membranes and produce efficient chitosan-degrading enzymes. Chitosan modulates gene expression and activates fungus development (e.g., conidiation) and expression of BCF pathogenicity factors such as serine proteases involved in the degradation of host barriers. Chitosan is also essential for virulence of human pathogen *Cryptococcus neoformans* [8]. Chitin and Chitosan have also been linked with the immune response of plant [3] and animal hosts [9,10]. Chitin and host enzymes with chitinase activity can also modify the inflammatory response crucial in human pathologies such as asthma. Chitosan gene targets have been studied using two model fungi: baker´s yeast (*Saccharomyces cerevisiae*) and the filamentous fungus *Neurospora crassa*. In both organisms, reactive oxygen species (ROS) and plasma membrane seem to be key players in the mode of action of chitosan.

The use of *omics*-derived technologies has a large potential to discover gene targets of chitosan in fungal pathogens. This could be a fundamental step to develop chitosan as an antifungal. In the future, chitosan could also help to modulate gene functions of beneficial microbes, such as BCF. This might improve their plant growth promotion and biocontrol capabilities. In this special issue contribution, we will revise molecular, cell and agronomical approaches together with *omics* techniques to fully understand the multimodal action of chitosan in agrobiotechnological and health applications.

## 2. The Antifungal’s Crisis: Fungi and Yeast as Human Pathogens; Chitosan as New Alternative to Treat Fungal Infections

The incidence of microbial infections in humans has been dramatically rising over the last few decades. This is mainly due to the increasing number of patients suffering from immunosuppressive infections or diseases, such as AIDS or leukemia [11,12,13,14,15] or the immunosuppressive side effects of cancer chemotherapeutics [16]. Furthermore, advanced and sophisticated medical treatments that suppress the immune system of severely compromised patients prolong their lives at the costs of an elevated risk for microbial infections even with low-virulence organisms [17,18,19].

Fungi and yeast are responsible for an increasing number of infections in immunocompromised patients. *Candida* spp. are the cause of infections in 50% of HIV+ and 90% in AIDS patients [20]. In the US, therapies against yeast and filamentous fungi infections cost about 2.6 billion dollars yearly. Of these, *Candida* spp. infections cost about 1.8 billion dollars, and similar figures are valid for Europe [20]. *Aspergillus fumigatus* infections are 100% fatal if untreated [21].

Pathogenic microorganisms becoming resistant to conventional drugs are sharply increasing due to intrinsic primary resistance or the development of secondary resistance as a result of long-term antimicrobial therapies [22]. The generation of new antifungal drugs encounters major obstacles because host and invading fungal pathogens share a large number of the cell, physiological and metabolic potential targets. Therefore, new and cost-effective strategies to combat fungal infections are essential. For this purpose, novel antimycotics with unique fungal targets and no side effects for the infected host are urgently needed [23,24].

Chitosan is a natural compound with a demonstrated antimicrobial activity [25]. Chitosan inhibits growth of filamentous fungi and yeast human pathogens [5,6]. However, to develop chitosan as an antifungal treatment, a full understanding of its mode of action is necessary. Recent studies have revealed relevant information on specific protein or gene targets in filamentous fungi and yeast. These studies open new possibilities to develop chitosan as a promising antifungal. In addition, chitosan is compatible with mammalian cells (COS7; monkey cell line), including somatic (HEK293 cell line) and immune human cells such as lymphocytes [6]. Very recently, self-assembled chitosan–miRNA nanocomplexes have shown successful downregulation of target mRNA expression in cancer cells [26]. Chitosan therefore offers a promising non-viral platform for gene therapy.

## 3. Chitosan and Chitooligosaccharides: Abundant, Biologically Active Biopolymers with Antifungal Properties

Chitosan is a polymer of glucosamine obtained by partial chitin *N*-deacetylation [27]. Chitosan is generated by chitin deacetylases, in the cell wall of zygomycetes [28,29]. Commercial chitosan is mainly obtained by chemical deacetylation of crustacean chitin from shellfish waste [27], but can also be produced by microbial/enzymatical methods [30]. The positive charge of chitosan confers unique physiological and biological properties on this polymer, with great potential in a wide range of industries such as pharmacology, medicine and agriculture [27].

Since Allan and Hadwiger (1979; [25]) first showed the fungicidal effect of chitosan, it has attracted a great deal of research attention, and several studies have been undertaken over the last thirty years on the chitosan sensitivity of different fungi with the purpose of understanding chitosan’s mode of action. Fungal growth inhibition by chitosan was associated with morphological and ultrastructural alterations of hyphae [31,32,33,34,35]. Chitosan not only inhibits hyphal growth but also spore germination [7,36]. Biological activity of chitosan depends on its molecular weight, deacetylation degree (DD) and pH of the solution. Stössel and Leuba (1984; [37]) found that the DD of the chitosan and the type and pH of the growth medium could affect the bioactivity of the biopolymer. The results of Hirano and Nagao (1989; [34]) indicated that low-MW chitosans were more effective than high-MW chitosans in inhibiting mycelium growth, which was confirmed in later studies [38,39]. Chitosan biological activities may depend not only on the degree of polymerization and acetylation, but also on a specific pattern of acetylation (PA). Chitosan oligomers with novel and defined PA have recently been produced by enzymatic deacetylation of chitin oligomers [40].

The nutritional status of the environment largely determines the antifungal effect of chitosan. Carbon (C) and nitrogen (N) limitation increase the antifungal activity of chitosan against filamentous fungi and yeast [6]. The C source also plays a key role since lactate in the growth medium increases *N. crassa* sensitivity to chitosan respect to glucose.

**Figure 1:**
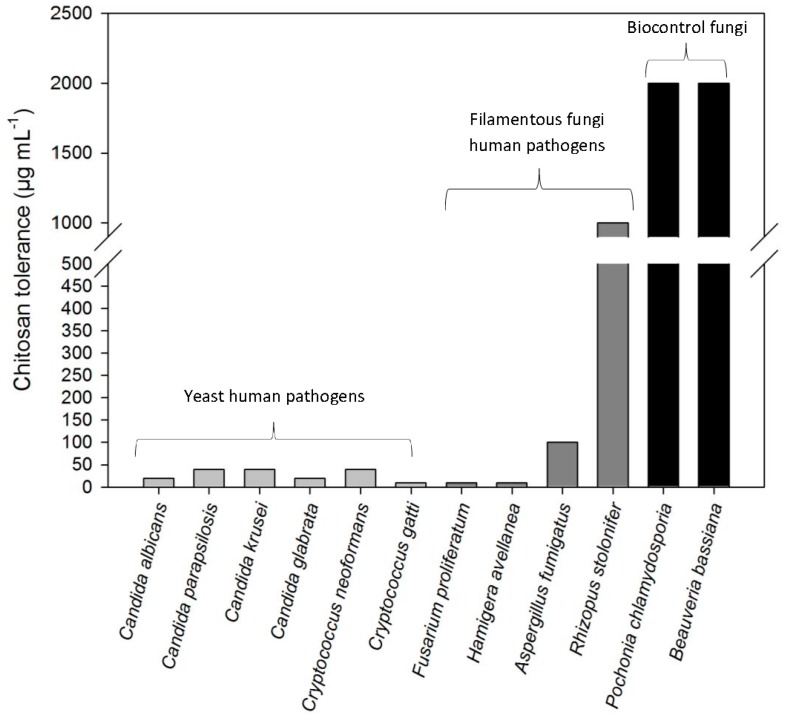
Filamentous fungi and yeast show large variability in chitosan sensitivity. Chitosan sensitivity is represented by the MIC (µg·mL^−1^) when the fungi are grown with chitosan in liquid media (unpublished and data from [6,7]).

**Figure 2:**
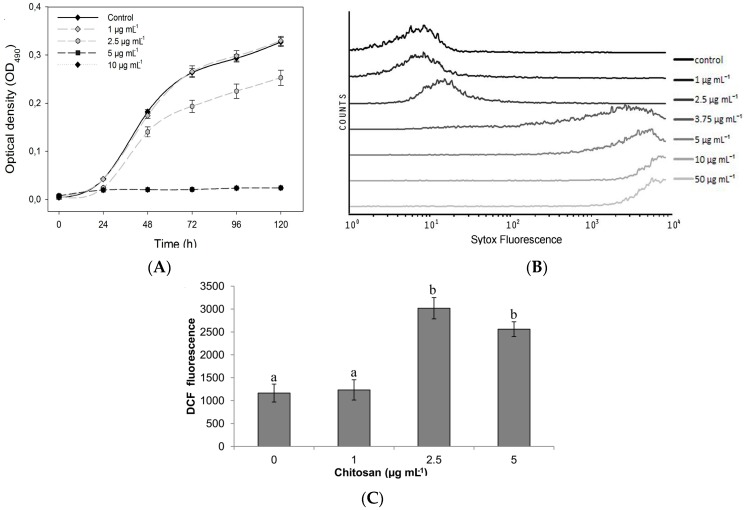
Chitosan inhibits growth of filamentous fungi (e.g., *N. crassa*) at low doses (**A**) by means of plasma membrane permeabilization which can be detected by flow cytometry (**B**); This process is preceded by early induction of intracellular oxidative stress detected by 2′–7′ dichlorofluorescein diacetate (DCF). Letters indicate statistical differences (*p* < 0.05) between chitosan concentrations (**C**); Graphs obtained from Lopez-Moya *et al.* 2015.

Chitosan inhibits growth of *Candida* spp. strains resistant to currently used antifungals such as fluconazole and amphotericin B. Chitosan displays high antifungal activity on *C. glabrata* (azole resistant), *C. parasilopsis* (fluconazole and echinocandin resistant) and *C. kruseii* (fluconazole resistant) even at low concentrations (20–40 µg·mL^−1^; Figure 1). Chitosan also inhibits bacterial growth [41,42,43] damaging their plasma membranes [41,44]. Chitosan permeabilizes the plasma membrane (Figure 2) and kills cells of the model fungus *Neurospora crassa* in an energy-dependent manner [1]. This is a very fast process that only takes a few seconds and varies depending on the cell type, leading to cell death from 4 min in conidia to 40 min in germlings and hyphae. Therefore different cell types vary in their sensitivity to chitosan. When cell energy is blocked, fluorescently labeled chitosan can only be detected in the conidial surface and not inside the cell [7]. The differential sensitivity to chitosan of different cell types observed for *N. crassa* confirms similar results previously obtained for plant pathogenic fungi [7] and human pathogenic fungi and yeast [6]. The energy requirement for chitosan to permeabilize plasma membrane and kill cells could be explained by an apoptotic response after membrane damage caused by chitosan. Sodium azide, used to test chitosan effect in the absence of adenosine tri-phosphate (ATP), is a well-known inhibitor of cytochrome oxidase, blocking the mitochondrial respiratory chain and thus ATP production in the mitochondria. Sodium azide not only inhibits ATP but also ROS production in *S. cerevisiae*, thus inhibiting apoptosis [45]. Thus, pre-treatment with sodium azide may be blocking the apoptotic response after cells detect membrane damage caused by chitosan, thus enhancing their tolerance.

## 4. Transcriptomics and Chemogenomics Reveal Key Gene Targets to Chitosan for Filamentous Fungi and Yeast

**Figure 3:**
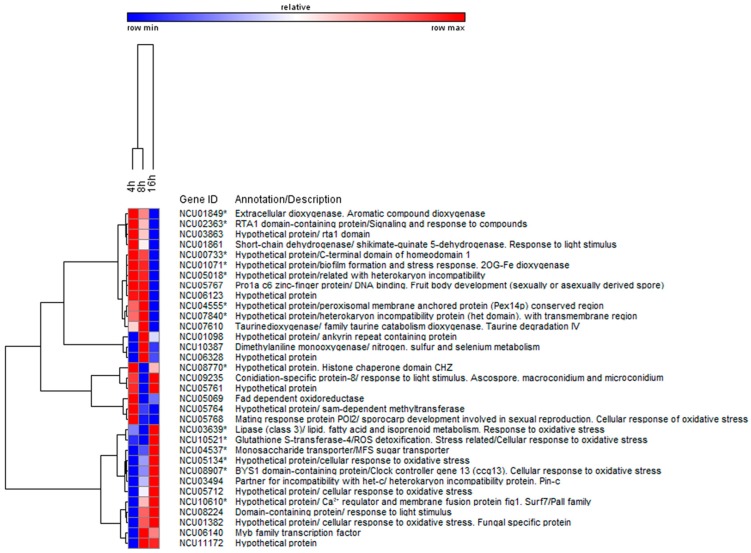
Hierarchical cluster analysis of a set of 33 genes involved in chitosan response to chitosan with an early and late induction in response to chitosan. Elaborated from Lopez-Moya *et al.* 2016 using data from GEO Series accession number GSE75293 [48]. This figure was created with the software GENE-E from Bioconductor repository [49].

Transcriptomics reveals the importance and relevance of genes related with plasma membrane, respiration, ATP production and mitochondrial organization in the response of *S. cerevisiae* to a 5.7 kDa chitooligosaccharide (COS) [46]. Using a chemogenomics and transcriptomic approach, five genes (*arl1*, *bck2*, *erg24*, *msg5* and *rba50*) provide COS resistance when overexpressed or increased sensitivity as deletion strains [46]. These genes have important roles in signaling pathways, cell membrane integrity and transcription regulation. *S. crevisiae arl1* deletion strain is sensitive to COS. On the contrary, when this gene is overexpressed, yeast becomes resistant to this antifungal. ARL1 encodes a G-protein (GTPase) member of the Ras superfamily. This protein is conserved in a wide number of organisms presenting 65% homology with this gene in humans. ARL1 is a plasma membrane protein associated with signaling pathways acting as a sensor and modulating membrane homeostasis. This protein could play a determinant role in the process of signaling during plasma membrane permeabilization of yeast by COS. A new transcriptomic study has recently been performed with the aim of investigating the mode of action of chitosan on filamentous fungi. In this study, chitosan induces changes in expression of 5% of *N. crassa* genes [47]. Previous studies demonstrate the determinant role of specific genes related with oxidative stress metabolism and with plasma membrane homeostasis in the response of *N. crassa* to chitosan. Transcriptomic data reveal a set of 33 main genes involved in the response of *N. crassa* to chitosan (Figure 3). Some of them are related with a quick response to chitosan (early upregulated) such as an extracellular dioxygenase related with response to oxidative stress or proteins involved in signaling mechanisms and response to chemical compounds such as an RTA1 domain-containing protein. Other genes show a late response to chitosan indicating its relevance in mechanisms of adaptation to this compound, such as those related with plasma membrane homeostasis.

A determinant role in *N.* crassa response to chitosan was found for a class III lipase involved in plasma membrane lipid replacement (NCU03639 encoded) and a monosaccharide transporter (NC04537 encoded), both proteins located in the plasma membrane. When their encoding genes are deleted, fungal sensitivity to chitosan increased with respect to *wt.* Transcriptomics reveals induction of oxidative stress metabolism genes, such as a glutathione transferase (NCU10521), when chitosan affects growth of *N. crassa*. The glutathione transferase could play a determinant role in ROS detoxification, since when its encoding gene was deleted, *N. crassa* sensitivity to chitosan increased.

## 5. Physiological Mode of Action of Chitosan on Sensitive and Resistant Fungi

Chitosan was found to enter most cells of chitosan-sensitive fungi, but could not do so (less than 30% conidia) in chitosan-resistant fungi. Plasma membrane of chitosan-sensitive fungi contains more polyunsaturated fatty acids (higher fluidity) than that of chitosan-resistant fungi. Therefore, plasma membrane permeabilization by chitosan could be dependent on membrane fluidity. This was confirmed using a free fatty acid desaturase deletion strain of *N. crassa* with reduced polyunsaturated fatty acid composition which exhibited increased resistance to chitosan *vs. N. crassa* wild-type [2]. Chitosan sensitivity can therefore be modified by altering plasma membrane fluidity. Phospholipid head groups have previously been proposed as the possible target for chitosan binding [32], so the different chitosan sensitivities of different fungi have been speculated to be due to differences on phospholipid head composition [35]. However, no correlation between phospholipid composition and chitosan sensitivity was found for four fungi tested [2]. Similar relative amounts of negatively charged phospholipids were found for chitosan-sensitive (*N. crassa* and *Fusarium oxysporum*) and resistant fungi (*Pochonia chlamidosporia* and *Beauveria bassiana*). The same was found for ergosterol, an important component of fungal plasma membranes, contributing to their rigidity, stability and resistance to physical stresses [50], which did not show correlation with chitosan sensitivity in fungi. Glycoinositolphosphorylceramides (GIPCs) are localized in the plasma membrane of fungi, plants and protozoa, but have not been found in cells or tissues of mammals or other higher animals [51]. GIPCs have also previously been proposed as possible targets for chitosan binding [52]. This could explain why chitosan is not toxic to mammals and higher animals [53,54]. However, no correlation was found between GIPC profiles and differences in chitosan sensitivity in fungi [2]. Both phospholipid heads and GIPCs may be the targets for chitosan binding. This was confirmed by steady-state fluorescence anisotropy measurements on artificial membranes that showed that chitosan binds to negatively charged phospholipids. This binding would induce an increase in rigidity in the membrane regions where it attaches, which would in turn induce membrane permeabilization by variations in fluidity between different regions. Chitosan would then be acting in a similar way to other positively charged antimicrobial peptides [55,56]. This permeabilization was greatest in membranes containing more polyunsaturated lipids where the change in fluidity after chitosan binding would be to a large extent. Hence, sensitivity of a fungus to chitosan could be predicted based on its plasma membrane composition. Furthermore, increasing plasma membrane fluidity would make fungi more sensitive to fungicides with a mode of action similar to that of chitosan.

## 6. Chitosan as a Versatile Compound Compatible with Biocontrol Fungi

Chitosan can be combined with beneficial fungi tolerant to this compound, such as biocontrol fungi (BCF). BCF have a large capability to degrade chitosan using it as a nutrient source [7]. Nematophagous fungi, including *P. chlamydosporia*, are able to grow with high doses of chitosan. The genome of this fungus [57] shows an expansion of hydrolase enzyme families reflecting its multitrophic (saprotrophic, endophytic, nematophagous) behavior. *P. chlamydosporia* encodes a large number of glycosyl hydrolases, including chitinases (GH18), chitosanases (GH75) and chitin deacetylases (CE4). These enzymes could be involved in the large capacity of this fungus to degrade chitosan. Proteomics reveals that chitosan induces expression of VCP1 serine protease, a putative pathogenic factor of *P. chlamydosporia* to nematode eggs [58]. Chitosan also induces expression of VCP1 and SCP1 (serine carboxypeptidase) in appressoria of *P. chlamydosporia*, thereby enhancing infection of root-knot nematode eggs [59]. Chitosan also affects BCF sporulation (*P. chlamydosporia* and *B. bassiana* [7].

## 7. Conclusions

**Figure 4:**
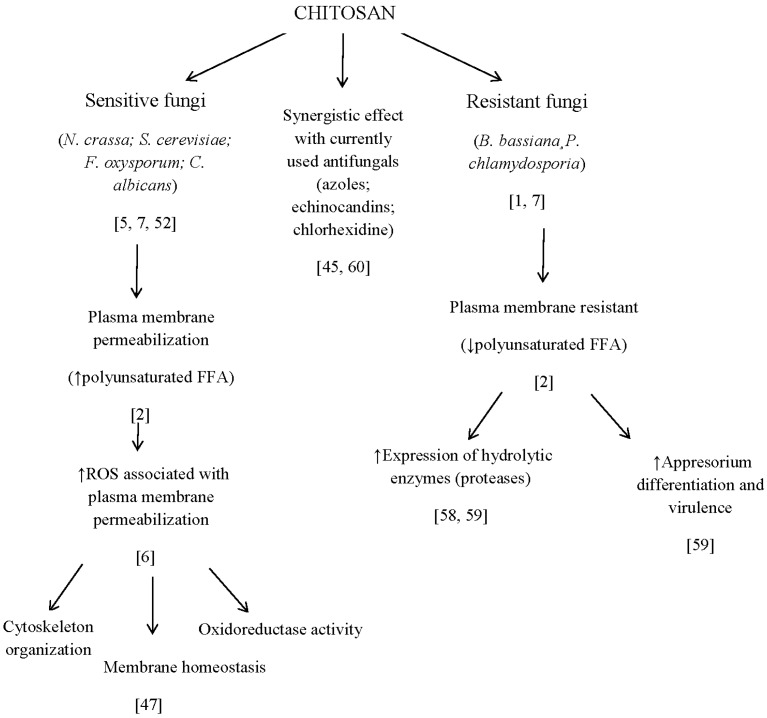
Conceptual diagram of chitosan as an antifungal and gene modulator.

Chitosan is a promising compound to control important fungal human pathogens. This will help to reduce emergence of antifungal resistant strains, especially in hospitals. Chitosan acts on the plasma membrane of sensitive fungi (rich in polyunsaturated free fatty acids; Figure 4), thereby increasing intracellular oxidative stress and ultimately leading to membrane permeabilization and cell death. Recent transcriptomic studies have identified plasma membrane homeostasis and oxidative metabolism genes determinant in the response of the model fungus *N. crassa* to chitosan.

Biocontrol fungi such as *P. chlamydosporia* have a low content of polyunsaturated free fatty acids in their plasma membranes and are resistant to chitosan. Genome sequencing of *P. chlamydosporia* reveals a wide gene machinery to degrade and assimilate this polymer. Moreover, chitosan increases sporulation and enhances parasitism of plant parasitic nematodes by the fungus. Therefore, chitosan can also be used in agrobiotechnological applications. *Omics* is a very useful tool for understanding the chitosan mode of action, thus allowing its use in biomedicine and agrobiotechnology.
